# The daily impact of COVID-19 in gastroenterology

**DOI:** 10.1177/2050640620920157

**Published:** 2020-04-11

**Authors:** Fernando Magro, Candida Abreu, Jean-François Rahier

**Affiliations:** 1 Department of Biomedicine, University of Porto, Porto, Portugal; 2 Department of Gastroenterology, Centro Hospitalar São João, Porto, Portugal; 3 MedInUP, Center for Drug Discovery and Innovative Medicines, Porto, Portugal; 4 Infectious Diseases Service, Centro Hospitalar São João, Porto, Portugal; 5 Instituto de Inovação e Investigação em Saúde (I3s), Grupo de I&D em Nefrologia e Doenças Infeciosas, Instituto Nacional de Engenharia Biomédica (INEB), Department of Medicine, Faculty of Medicine, University of Porto, Portugal; 6 Department of Gastroenterology, Université Catholique de Louvain, Yvoir, Belgium *Fernando Magro and Candida Abreu contributed equally to this work.

**Keywords:** Gastroenterology, inflammatory bowel disease, endoscopy, epidemiology, immunology

## Abstract

A new strain of coronavirus, called SARS-CoV-2, emerged in Wuhan, China, in December 2019, probably originating from a wild-animal contamination. Since then, the situation rapidly evolved from a cluster of patients with pneumonia, to a regional epidemic and now to a pandemic called COrona VIrus Disease 2019 (COVID-19). This evolution is related to the peculiar modes of transmission of the disease and to the globalization and lifestyle of the 21st century that created the perfect scenario for virus spread.

Even though research has not evidenced particular susceptibility of inflammatory bowel disease (IBD) patients to SARS-CoV-2 infection, immunosuppressive and immunomodulatory treatments were considered potential risk factors. In this context, initiating treatments with these agents should be cautiously weighted and regular ongoing treatments shall be continued, while the dose of corticosteroids should be reduced whenever possible. Due to the increased risk of contamination, elective endoscopic procedures and surgeries should be postponed and IBD online appointments shall be considered. IBD patients shall also follow the recommendations provided to the general population, such as minimization of contact with infected or suspected patients and to wash hands frequently.

In the absence of effective treatments and vaccines, this pandemic can only be controlled through prevention of SARS-CoV-2 transmission with the main objectives of providing patients the best healthcare possible and reduce mortality.

## Introduction

Coronaviruses are a large group of viruses that affect both animals and humans. For a long time, these viruses were not a matter of concern since they were solely recognized as the cause of common cold.^1,2^ This situation changed in 2002–2003, when severe acute respiratory syndrome (SARS) emerged as a consequence of human infection by a virus of this family, named as SARS coronavirus (SARS-CoV).^1^ At that time, SARS affected 32 countries and more than 8000 people, causing 919 deaths (case fatality rate of 11%). A worldwide epidemic was halted through the efforts of the World Health Organization, which responded rapidly to this threat by issuing a global alert, rigorous local containment efforts and a stern warning against unnecessary travel to affected areas. Since 2003, the medical literature has not reported SARS-CoV circulation in human populations.^3^

Ten years later, the Middle East respiratory syndrome coronavirus (MERS-CoV) was first isolated, in Saudi Arabia, from a man who died from acute pneumonia and renal failure. The infection was responsible for 2494 cases and 858 deaths in 27 countries (case-fatality rate of 34.4%).^1^ The third coronavirus epidemic was first reported in Wuhan, China in December 2019, where a cluster of patients, with pneumonia of unknown cause, was identified. Zhu and collaborators isolated a novel coronavirus from the bronchoalveolar-lavage fluid of three of these patients.^4^ The researchers used next-generation sequencing and polymerase chain reaction (PCR) to characterize the virus which falls within the subgenus *Sarbecovirus* of the genus *Betacoronavirus*. This new strain, called SARS-CoV-2, has cytopathic effects (structural changes in host cells)^4^ and, like SARS-CoV, probably originated from wild animals. The homology between coronavirus isolated from bats, snakes and pangolins, and SARS-CoV-2 isolated from infected humans makes these animals potential carriers.^5^

The first patients infected by this new virus, in Wuhan, caused the epidemic known as COrona VIrus Disease 2019 (COVID-19). From Wuhan, a massive human-to-human transmission occurred^3^ taking advantage of the intensive national and international travel during Chinese New Year. The infection rapidly spread all around the World, with cases identified across the six continents.

## The virus and infection

The basic reproductive number (R0) of SARS-CoV-2 ranges from 2 to 5.5,^6^ among different prediction models, which is much higher than those of SARS (R0 ≈ 3)^7^ and MERS (R0 < 1).^6^ The spread of SARS-CoV-2 illustrates two important modes of disease transmission in the modern era. The first modus is local transmission, at each local epicentre, while the second is transmission via international travellers which favours the global spread of the infection, fuelling the pandemic of COVID-19. Large outbreaks were reported in closed communities and hospitals, raising the possibility of ‘superspreading’ events, a situation observed in previous coronavirus outbreaks.^8^ The transmission of the virus occurs person-to-person, primarily through respiratory droplets, but also by contact and fomites, nosocomial transmission^9^ and possible aerosol and faecal–oral transmission.^10^ Person-to-person transmission is being evidenced by family clusters of pneumonia associated with the 2019 novel coronavirus.^11^ Research also indicates that SARS-CoV-2 can have pre-symptomatic transmission during the prodromal period^3,11^ and that viral shedding appears to occur in individuals with minor clinical manifestations.^12^ Data concerning transmission during the disease course is scarce; a small study, among 25 asymptomatic COVID-19 cases, considered a communicable period up to three weeks.^13^ All these particularities contribute to the extensive community transmission that is being registered. As SARS-CoV-2 can remain viable and infectious in aerosols (for hours) and on surfaces (up to days), aerosol fomite transmission is plausible and is a key aspect to consider while developing pandemic mitigation plans.^14^ Vertical transmission was not yet proved.^15^

This virus has affinity for cells of the lower respiratory tract, causing non-life-threatening or severe pneumonia.^3^ The patterns of the clinical course of infection are divided into: (a) mild upper respiratory tract illness; (b) non-life-threatening pneumonia; (c) and severe pneumonia, with acute respiratory distress syndrome (ARDS), which begins with slight symptoms (for 7–8 days) that rapidly progress to ARDS, requiring support ventilation. The average incubation period of SARS-CoV-2 is 6.4 days, ranging from 0 to 24 days.^15^ The elderly and those with comorbidities (cardiovascular disease, diabetes, chronic respiratory disease, hypertension and cancers) are at risk of more severe infection and present higher case fatality rates (10.5, 7.3, 6.5, 6.0 and 5.6%, respectively) than those without comorbidities (0.9%).^16^ SARS-CoV-2 may also cause damage to other organs such as heart, liver and kidneys, as well as to organ systems such as the blood and the immune system.^16–18^ Patients eventually die of multiple organ failure, shock, acute respiratory distress syndrome, heart failure, arrhythmias, or renal failure.^18,19^ It seems that groups with relative immunosuppression, such as very young children, pregnant women and HIV patients are not at higher risk of complications (British HIV Association (BHIVA) https://www.bhiva.org/comment-on-COVID-19-from-BHIVA). Particular attention should be devoted to smokers, in whom there is evidence of high susceptibility to SARS-CoV-2.^20^ Tobacco increases the gene expression of the angiotensin converting enzyme, the binding receptor for this virus.^21^

## Endoscopy units and disinfection

**Figure 1.**
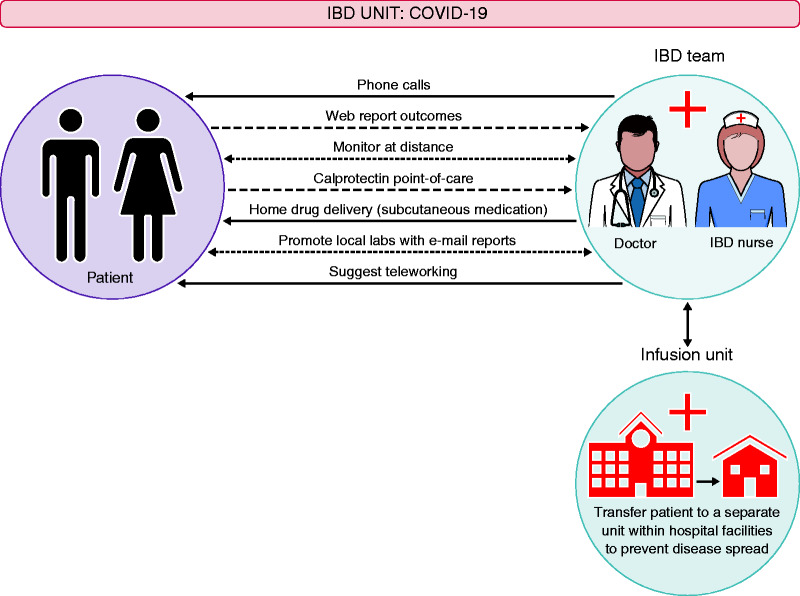
Protection level in an endoscopy unit in a COVID-19 infection area

The potential of transmission of SARS-CoV-2 by objects or surfaces and by the faecal–oral route is a major concern in endoscopy units. On the other hand, exhaustion, burnout and inexperience in a crisis, like the one we are experiencing, increase the possibility of human errors.^22^ Endoscopy should still be regarded as a risky procedure that can expose endoscopists to potential contamination. Viruses, in particular, can spread via the airborne route during endoscopy aerosolization. This is a potential risk concerning SARS- CoV-2 virus as transmission may be favoured during endoscopy. In this context, endoscopy should be performed in a negative-pressure room and the staff of the endoscopy department should follow standardized precautions. Personal protective equipment (PPE) should be used according to low or high-risk dressing.^23^ In addition, the American Society of Gastroenterology Endoscopy (ASGE) guidelines for infection control in endoscopy^23^ and the European Society of Gastrointestinal Endoscopy (ESGE) guidelines^24^ must be strictly followed. All the equipment, including endoscopes and reusable accessories, should be reprocessed with a standardized reprocessing procedure. The disinfectants, used for this purpose, must be bactericidal, mycobactericidal, fungicidal and virucidal (against enveloped and non-enveloped viruses). SARS-CoV-2 is easily inactivated by many commonly used disinfectants and no additional approach should be implemented to clean and disinfect the endoscope.^25^ Regarding the endoscopy-centre environment, UV irradiation and ozone treatment are recommended for the cleaning and sterilization of air and surfaces, such as endoscopic equipment, office tables and the walls of the examination room. As surfaces might be a possible source of contamination and can lead to infection, cleaning all the surfaces in the procedure room is a key point. Endoscopy-centre furniture and floors can be heavily contaminated in the case of SARS-CoV-2 infection and should be thoroughly disinfected after each procedure. Chlorine-containing detergent is recommended for daily floor cleaning. Due to the risk of aerosolization, in the absence of negative pressure the room should be kept empty for at least 1 h, and for 30 min in the case of negative-pressure rooms, before the procedure.^22^ In outbreak areas, only emergency endoscopies should be performed. These exceptions can include cases of acute gastrointestinal bleeding, presence of foreign bodies in the gastrointestinal tract and acute suppurative cholangitis. In pandemic areas the basic protection requirements of the medical staff in the endoscopy centre should reach Biosafety level 2 (the wearing of disposable gowns, N95 masks, goggles, caps and shoe covers during endoscopy) for all kinds of GI endoscopic procedures. Biosafety level 3 (PPE, respirators, plus negative-pressure rooms, due to the aerosolization risk of SARS-CoV-2 virus during endoscopy) protection is required for all endoscopic procedures in SARS-CoV-2 infected or suspected patients, and for those with very high risk of potential exposure. The criteria for the selection of the adequate biosafety protection level in an endoscopy unit are outlined in Figure 1.

## Immunosuppression and COVID-19

Data collected from more than 200,000 IBD patients from IBD referral centres in China do not evidence a particular susceptibility of patients on immunosuppressants or biologics to SARS-CoV-2 infection,^19^ and as a consequence, none of the three largest tertiary IBD centres in Wuhan published any report on this topic.^26^ Nevertheless, in February the Chinese IBD Society made practical recommendations concerning this group of patients and listed the potential risk factors for SARS-CoV-2: immunosuppressive therapy, diabetes, hypertension, older age, malnutrition and pregnancy. Until now, research could not identify reasons to stop current treatment in stable patients, including those on anti-TNF, vedolizumab, ustekinumab, immunomodulators (azathioprine, methotrexate), JAK-inhibitors and salicylates. For patients with active/severe COVID-19 infection, the risk must be individualized. However, all biologics have long half-lives and will be in circulation during the infection period, with a slow and reduced impact on the IBD clinical situation. On the other hand, the interruption of treatments with corticosteroids, methotrexate or JAK-inhibitors can induce a fast decrease of their serum levels, with possible reflex in the clinical situation. Particular attention should be paid to corticosteroids, especially in doses above 20 mg. Even though current evidence does not suggest the switch from infliximab to adalimumab (to avoid hospital visits), this hypothesis should be considered in high pandemic areas where the local infusion centre cannot afford appropriate treatments anymore. However, these situations must be carefully followed since elective switching from infliximab to adalimumab is associated with a loss of tolerance and efficacy within 1 year.^27^

During the SARS-CoV-2 pandemic, all elective endoscopic procedures and surgeries should be postponed, and infection screening is recommended before emergent surgeries. Also, to minimize the absence to regular follow-up appointments, IBD online appointments shall be considered. The advice regarding IBD patients on immunomodulators and biologics are summarized in Table 1.

**Table 1.** Advice regarding patients on immunomodulators and biologics.

General principles
• Having IBD or liver disease is not a risk factor for COVID-19; • the COVID-19 infection risk is similar in Crohn’s disease and ulcerative colitis; • patients should stay on their therapies and stay in remission.
General measures
• Avoid contact with infected people; • avoid touching your eyes, nose or mouth with unwashed hands; • clean hands often by washing them with soap and water, for at least 20 seconds, and/or using an alcohol-based hand sanitizer that contains 60–95% alcohol.
Travellers
• IBD patients shall be discouraged from travelling; • defer all cruise-ship travel worldwide; • avoid crowded places and long flights; • avoid close contact with people with acute respiratory infection and wash hands frequently.
Medication and procedures
• Keep ongoing immunomodulators; • keep ongoing biologics; • avoid prednisolone; • reduce corticosteroids whenever possible; • use masks in IV infusions units; • postpone elective endoscopic procedures and surgeries; • postpone the start of immunomodulators and biologics unless active disease should prompt initiation.
Close contact with someone with proven COVID-19 infection
• Follow national recommendations, as any other person; • effects of treatment suspension are scarce; • do not stop medication.
Patients in clinical trials
• Try to postpone the follow-up visit with the sponsor; • phone calls and patient report outcomes (web-system); • home drug delivery; • local laboratories with email reports.

## IBD unit’s strategy during COVID-19 infection

**Figure 2.**
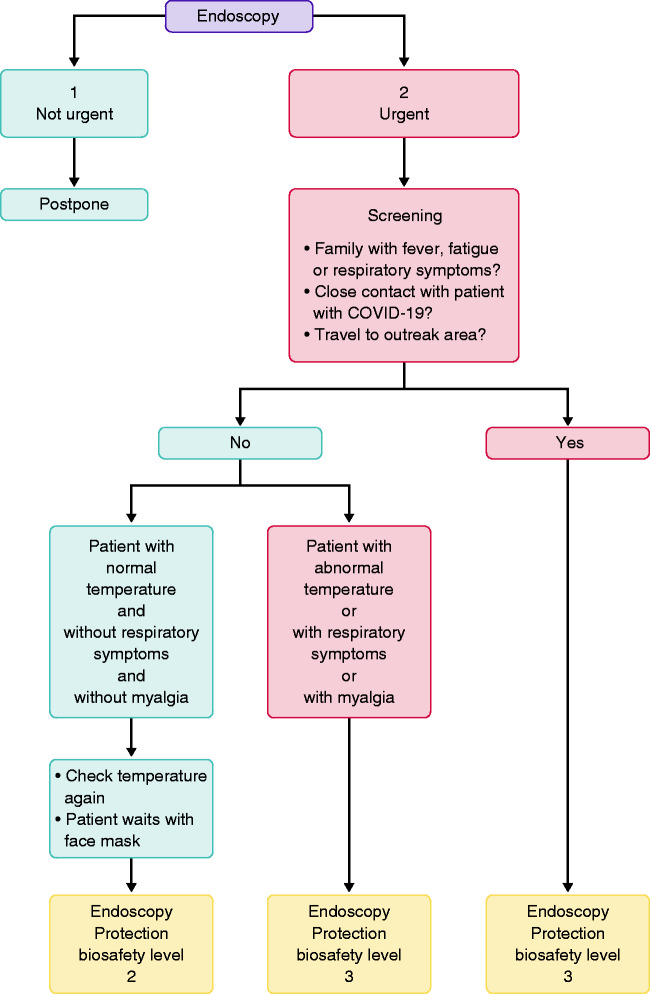
IBD unit’s strategy during COVID-19 infection.

In IBD units, compulsory temperature checks should be performed daily in the entire staff before entering the work area. Hand hygiene should also be obligatory, with recommendations to follow the ‘six- step hand-washing method’. Virtual clinics or online consultancy should be actively promoted. Patient-reported outcomes, simple questionnaires on symptoms, concomitant medication and questions are determinant and will be the preferential means of communication. Visit phone calls and online prescriptions are also important tools for communication. All points of care should be implemented, particularly home faecal calprotectin, which will be particularly useful in cases of gastrointestinal symptoms. Wherever any doubt considering COVID-19 diagnosis remains, testing for the virus is mandatory. The strategy is summarized in Figure 2.

## Liver and SARS-CoV-2

COVID-19 is a systemic disease that affects a wide spectrum of tissues and cell types. Liver cells and bile duct cells express ACE2. However, the ACE2 expression of bile duct cells is much higher than that of liver cells, but comparable to that of alveolar type 2 cells in the lung. Efforts to isolate the virus from the liver have not succeeded. In patients surveyed so far, liver biochemistry findings are common, and transaminases are elevated in 14–53% of cases.^28^ In more severe cases, research evidenced a possible correlation between disease severity and higher frequency of elevated transaminases.^16^ Though elevated liver transaminases suggest that hepatic dysfunction is common, serum Gamma-glutamyl transferase (GGT) is elevated in 54%, while elevated alkaline phosphatase levels is rare.^28^ In a large cohort of Chinese patients, bilirubin was elevated in 10% of cases. However, fatality as a result of liver failure has not been reported so far. Liver injury in severe COVID-19 patients was significantly higher than that in mild patients.^16^ At this point, a better picture of the impact of COVID-19 infection in patients, with a pre-existing liver disease or on immunosuppression starts to emerge. So far, the data suggest that patients with a pre-existing liver disease (such as chronic hepatitis B infection or liver cirrhosis) do not have an additional risk. Current research could not evidence that COVID-19 causes a more severe disease in immunosuppressed patients, such as liver-transplant patients or patients treated with immunosuppressants (like those with auto-immune hepatitis). The Bergamo experience (in which approximately 700 children received a liver transplant, three in the last two months) showed that, among about 200 transplant recipients, none has developed clinical pulmonary disease, despite three testing positive for SARS-CoV-2.^29^ Considering their immunocompromised status, more intensive surveillance or individually tailored therapeutic approaches is needed for severe patients with COVID-19, with pre-existing conditions such as advanced liver disease. This is particularly important for the elderly with other comorbidities.

## Travellers

Travelling, for everyone, should be limited as much as possible. In case of essential travelling, visitors should take routine precautions when entering and leaving affected areas. These include: (a) avoiding close contact with patients with acute respiratory infection; and (b) washing hands frequently, especially after contact with infected patients or their surrounding environment.

When individuals have travelled to a pandemic area in the past 14 days and present fever, cough or difficulty in breathing, they should: (a) call the doctor to report recent trips and symptoms, before going to the emergency room or to the doctor’s office; (b) avoid contact with others; (c) not travel around; (d) cover the mouth and nose with a tissue or sleeve (not hands), when coughing or sneezing; and (e) wash hands with soap and water for at least 20 s. If soap and water are not available, use alcohol-based hand sanitizers.

## COVID-19 new developments

From the public-health perspective, the priority is now the development of antiviral therapeutics to stop the SARS-CoV-2 pandemic and of an effective vaccine to prevent future epidemics. Efforts are ramping up to the development of new therapeutics. Remdesivir, an investigational antiviral drug developed to treat Ebola and Marburg haemorrhagic fevers, showed activity against SARS-CoV-2, *in vitro*.^30^ This drug has been used in a few patients, on a compassionate-use basis, outside of a clinical trial setting. It is a nucleotide analogue and, like other drugs of that class, it disrupts nucleic acid production. Chloroquine phosphate and hydroxychloroquine (used for Lupus and rheumatoid arthritis) also showed activity against the virus.^31,32^ *In-vitro* studies evidenced that hydroxychloroquine was more potent than chloroquine in inhibiting SARS-CoV-2.^31^ Hydroxychloroquine treatment was significantly associated with viral-load reduction/disappearance in COVID-19 patients and its effect was reinforced by azithromycin, in a small sample size population.^29^ Lopinavir and ritonavir are also being studied in this context, but, in hospitalized adult patients with severe COVID-19, no benefit was observed beyond standard care.^33^ Another potential treatment option is coronavirus sera prepared from the blood of patients in convalescence (convalescent sera). Passive immunization is well established in viral infection prophylaxis for cytomegalovirus, hepatitis B virus and varicella-zoster virus. Although, the appropriate titre of convalescent sera antibody required for therapeutic efficacy against SARS-CoV-2 remains to be determined. From past experience, the success of this strategy is not guaranteed. For instance, research with MERS-CoV showed that sera from patients recovering from the disease did not contain sufficient antibody titres for therapeutic use.^34^

## Conclusions

Many gaps still remain in the overall knowledge on this ongoing pandemic. SARS-CoV-2 virus, although very similar to MERS-CoV virus, has much higher transmission rates, has inter-family transmission and can cause a severe and long disease. We cannot rely on previous knowledge and experience on influenza, although they are both respiratory viruses. The absence of an effective treatment, along with the lack of vaccines, are obstacles we need to go through. A reduction in mortality must definitely be a major objective.

Healthcare systems have serious difficulties while dealing with the disease because a huge number of patients need critical care, at the same time and for long periods of time. In the meanwhile, the economic and cultural life of our society is disrupted as a consequence of the pandemic. In this scenario, a huge effort to treat and prevent the disease is urgent.
